# Clinicopathologic comparisons of IgA nephropathy and IgA vasculitis nephropathy in children: a ten-year single-center experience

**DOI:** 10.3906/sag-2104-319

**Published:** 2021-10-21

**Authors:** QingXiao SU, YuHeng LIANG, Na WANG, ZhiYan DOU, ZanHua RONG, Xue ZHAO, Bo YU, YuXue WANG, XinLiang WANG

**Affiliations:** 1 Department of Peadiatrics, The Second Hospital of HeBei Medical University, Shijiazhuang 050000 China

**Keywords:** IgA nephropathy, IgA vasculitis nephritis, children, clinical and pathological features of kidney, anaphylactoid purpura

## Abstract

**Background/aim:**

To investigate the similarities and differences of renal clinical and renal pathology between IgA nephropathy (IgAN) and IgA vasculitis nephritis (IgAVN) in children.

**Materials and methods:**

A total of 237 children with IgAN and 190 children with IgAVN were included. The general conditions, clinical characteristics, final diagnosis, clinical and pathological classification of the children were intercepted at the time of admission, and the retrospective comparative analysis was carried out.

**Results:**

The results showed that the median course of disease in IgAN group was longer than that in IgAVN group (p = 0.02). Patients with IgAN had a significantly higher duration of infection than the patients with IgAVN (p = 0.03). The white blood cell count (WBC), hemoglobin (HGB) in IgAN group were significantly lower than that in IgAVN group (p = 0.02). The serum creatinine in IgAN group was higher than that in IgAVN group (p = 0.02). Patients with IgAN and IgAVN had statistically significant differences in pathological typing between clinical types: hematuria and proteinuria, nephrotic syndrome and chronic nephritis (p = 0.004).

**Conclusion:**

The clinical manifestations of IgAN and IgAVN were similar, but the onset of IgAN was hidden and the clinical manifestations were relatively serious. Renal pathology was mainly glomerulosclerosis and renal tubular atrophy. IgAVN was characterized by acute onset and good renal function. Renal pathology was dominated by endothelial hyperplasia and crescent formation. These differences did not support the hypothesis that the two diseases are the same.

## 1. Introduction

Anaphylactoid purpura, also known as IgA vasculitis nephritis (IgAV) was a systemic small vasculitis that can cause damage to multiple organs. Most of the cases occur in children aged 2–8 years, with more boys than girls. IgA vasculitis nephritis (IgAVN) was found in 30% to 60% of the cases [1]. It was characterized by varying degrees of hematuria and/or proteinuria, and the symptoms vary in severity, and there was no consistent relationship with the severity of extrarenal symptoms. In general, the prognosis of hematuria and mild proteinuria under the microscope was good. About 20% of IgAVN presents with nephrotic syndrome, and its prognosis was poor, which can lead to end-stage renal failure [1,2].

In the 2012 Chapel Hill conference, it was proposed to name anaphylactoid purpura directly as IgA vasulitis (IgAV), and it was pointed out that systemic vasculitis may develop from organ specific vasculitis. There was no difference between IgAVN and IgAN, and both were a component of IgAV [3]. The initiating factor of the pathogenesis of the two was the abnormal glycosylation of IgA1 [4], and the renal pathology of the two was the same [5,6]. The main pathological types of both IgAVN and IgAN were mesangial proliferative glomerulonephritis and focal proliferative glomerulonephritis [7]. At present, it is considered that the pathophysiology of IgAVN and IgAN was similar, and C3 can be seen in 75%–100% of IgAVN and IgAN patients [8]. This study was to investigate the similarities and differences of renal clinical and renal pathology between IgAN and IgAVN in children. 

## 2. Materials and methods

### 2.1. Research subjects

In this study, 237 children with IgAN and 190 children with IgAVN were included. Participants had been divided into IgAN group and IgAVN group. The diagnostic criteria, clinical classification, and pathological classification of IgAN and IgAVN were based on the standards of the Nephrology Group of the Pediatric Branch of the Chinese Medical Association [5,6]. This study was conducted in accordance with the Declaration of Helsinki and approved by the ethics committee of our hospital. 

### 2.2. Inclusion and exclusion criteria

Inclusion criteria: (1) Research subjects ≤18 years old; (2) The patients in this study were hospitalized in the Second Hospital of Hebei Medical University from January 2010 to December 2019, and were diagnosed with IgAN and IgAVN by renal biopsy. Exclusion criteria: (1) patients who have had other renal or nonrenal diseases; (2) patients whose data was incomplete.

### 2.3. Methods

Pathological grading: The pathological grading of IgAN was I–W according to Lee’s grading standard in 1982 [9]. The pathological grading of IgAVN was I–WI according to the grading standard of the International Study of Kidney Disease in Children (ISKDC) [10]. Oxford pathology classification was based on the newly revised Oxford classification method in 2017, namely MEST-C [11].

Clinical classification: According to the IgAN classification standard and IgAVN classification standard established by the Nephrology Group of the Pediatrics Branch of the Chinese Medical Association in 2016 [5,6]. According to whether the patients have hematuria, proteinuria, and nephritis symptoms, the patients were divided into hematuria and proteinuria type, acute nephritis type, nephrotic syndrome type, isolated hematuria type, chronic nephritis type, isolated proteinuria type and rapidly progressive nephritis type.

### 2.4. Main observation indicators

The demographic data, laboratory test items, clinical manifestations, and renal pathology of the patient at the first visit were included. Demographic data included age, sex, body mass index BMI (kg/m^2^). Laboratory examination items included white blood cell count (WBC), hemoglobin (HGB), platelets (PLT), 24-h urine protein, urine red blood cell count, blood urea nitrogen, serum albumin, total cholesterol, blood creatinine, immunoglobulin, complement and glomerular filtration rate. Renal pathology included light microscopy, immunofluorescence, the proportion of glomerular sclerosis, the proportion and type of crescent, loop necrosis, mesangial hyperplasia, endothelial hyperplasia, interstitial inflammation, tubule atrophy, IgM, IgG, IgA, C3, C1Q.

### 2.5. Statistical analysis

We used the software program SPSS 26.0 (IBM, Armonk, NY, USA) to conduct the statistical analysis. The continuous variables of normal distribution were expressed as mean ± standard deviation, the continuous variables of nonnormal distribution were expressed as median [interquartile range (IQR)], the categorical variables were expressed as frequency [percentage (%)]. For two comparisons, each value was compared by t-test when each datum conformed to normal distribution, while the nonnormally distributed continuous data were compared using nonparametric tests. The counting data were tested by chi-square test. A value of p < 0.05 was considered statistically significant.

## 3. Results

### 3.1. General characteristics

In this study, a total of 237 children with IgAN and 190 children with IgAVN were included. The screening process was shown in Figure. We screened out the cases preserved in our hospital, selected the patients who were treated in our hospital from January 2010 to December 2019, excluded the patients who were older than 18 years old and had incomplete data, and finally met the conditions of 237 patients with IgAN and 190 patients with IgAVN. In the IgAN group, there were 151 males and 86 females, the mean ± SD of age was 13.3 ± 2.3, the mean ± SD of BMI-SDS was 1.32 ± 0.25. In the IgAVN group, there were 106 males and 84 females, the mean ± SD of age was 13.2±3.1, the mean ± SD of BMI-SDS was 1.29 ± 0.38. The results showed that there was no significant difference in the age of onset and age distribution (<6 years; 6–12 years; >12 years), sex and BMI-SDS index between the two groups. 

**Figure:**
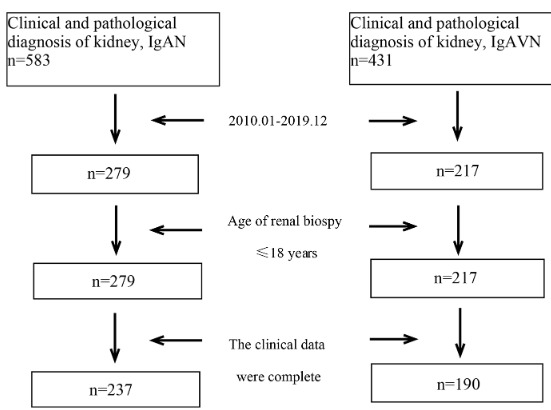
Flow chart of case screening in IgAN and IgAVN groups.

### 3.2. Clinical manifestations and laboratory tests

The median course of onset in the IgAN group was significantly longer than that of IgAVN. The IgAN group had a higher proportion of prodromal infection history than the IgAVN group (p = 0.03). There was no significant difference in family history, systolic blood pressure, diastolic blood pressure, and mean arterial blood pressure (MAP). The median courses of onset were listed in the Table 1. 

**Table 1:** Comparison of general clinical characteristics between IgAN group and IgAVN group.

Index	IgA nephropathy(n = 237)	IgA vasculitis nephritis(n = 190)	p
Age (year)	13.3 ± 2.3	13.2 ± 3.1	>0.05
>12	124 (52.3%)	100 (52.6%)	>0.05
6~12	96 (40.5%)	66 (34.7%)	>0.05
<6	17 (7.2%)	24 (12.7%)	>0.05
Sex			
Male	151 (63.7%)	106 (50.5%)	>0.05
Female	86 (36.3%)	84 (49.5%)	
Height-SDS	0.78 ± 1.28	0.82 ± 1.41	>0.05
BMI-SDS	1.32 ± 0.25	1.29 ± 0.38	>0.05
SBP-SDS	0.28 ± 0.12	0.31 ± 0.19	>0.05
DBP-SDS	0.33 ± 0.13	0.36 ± 0.18	>0.05
Median course of disease (months)	1.4 (0.63–8)	0.67 (0.33–3)	0.02
Family history	3 (1.3%)	8 (4.2%)	>0.05
History of precursor infection	134 (56.5%)	68 (35.8%)	0.03

The clinical classification of two groups of diseases in children were shown in the Table 2. There were significant differences in isolated proteinuria, nephrotic syndrome, acute nephritis and chronic nephritis between the two groups (p = 0.004). There were no statistically significant differences in hematuria and proteinuria, isolated hematuria, and acute progressive nephritis (p = 0.10).

**Table 2:** The clinical classification of two groups.

Index	IgA nephropathy(n = 237)	IgA vasculitis nephritis(n = 190)	p
Nephrotic syndrome type	81 (34.2%)	36 (19.0%)	0.004
Hematuria proteinuria type	84 (35.4%)	70 (36.8%)	0.004
Chronic nephritis type	54 (22.8%)	27 (14.2%)	0.004
Isolated hematuria type	9 (3.8%)	14 (7.4%)	>0.05
Acute nephritis type	5 (2.1%)	22 (11.6%)	>0.05
Isolated proteinuria type	3 (1.3%)	20 (10.5%)	>0.05
Acute progressive nephritis type	1 (0.4%)	1 (0.5%)	>0.05

There was no statistical difference in platelet count, glomerular filtration rate, urine red blood cell count, 24-h urine protein, serum globulin, serum cholesterol, serum complement C3, C4 and serum immunoglobulin between IgAN group and IgAVN group. The serum immunoglobulin IgA in two groups was higher than normal. The details were listed in the Table 3. 

**Table 3:** Comparison of laboratory indexes between two groups of children.

Index	IgAN(n = 237)	IgAVN(n = 190)	p
WBC (×109/L)	8.4 ± 4.5	10.2 ± 4.5	0.02
Hb (g/L)	126 ± 15.7	133.6 ± 16.15	0.02
PLT (×109/L)	290.0 ± 89.5	300.7 ± 84.6	>0.05
Hematuria (/uL)	3306 ± 6326.3	530.3 ± 822.5	>0.05
Proteinuria (g/24 h)	2.5 ± 2.3	1.9 ± 1.8	>0.05
Scr (umol/L)	70.7 ± 54.5	58.4 ± 17.6	0.02
eGFR (mL/min/1.73 m2)	146.2 ± 133.0	155.0 ± 130.6	>0.05
Alb (g/L)	32.6 ± 8.6	33.1 ± 8.7	>0.05
Chol (mmol/L)	4.9 ± 2.1	4.5 ± 1.6	>0.05
IgA (g/L)	2.3 ± 1.0	2.2 ± 0.9	>0.05
IgG (g/L)	7.1 ± 3.4	7.3 ± 4.4	>0.05
IgM (g/L)	1.2 ± 0.7	1.2 ± 0.6	/
C3 (mg/dL)	101.3 ± 25.8	102.5 ± 26.7	>0.05
C4 (mg/dL)	23.3 ± 8.9	22.8 ± 11.3	>0.05

Among the various clinical types of IgAN and IgAVN, the most common pathological types were type II and type III, followed by type I and type IV. No cases of type V in the pathological classification and type VI in IgAVN were observed in two groups. There were statistically significant differences between IgAN group and IgAVN group in pathological classification among the three different clinical types of chronic nephritis, hematuria and proteinuria and nephrotic syndrome [α^2 ^=136.56, p = 0.004, C^2 ^= 25.156, P = 0.008].

### 3.3. Renal pathology

The results showed that the renal pathology of the two groups showed no statistically significant differences in the proportion of glomerular sclerosis (including the proportion of spherical sclerosis and the proportion of segmental sclerosis), the degree of mesangial cell proliferation, the proportion of loop necrosis, and the proportion of interstitial cell infiltration (p = 0.08). The proportion of tubule atrophy in the IgAN group was significantly higher than that in the IgAVN group [132 (55.7%) vs. 17 (8.9%)], while the proportion of endothelial hyperplasia and crescent formation in the IgAN group was lower than that in the IgAVN group [41(17.3%) vs. 64 (34.7%), 56 (23.6%) vs. 71 (37.4%)], the difference was statistically significant. 

Immunofluorescence results showed that both groups had IgA immune complex deposition in the mesangial area, most of which are accompanied with C3 deposition, often accompanied with IgM deposition, a small part with IgG deposition, and rarely with Fibrogen and C1q deposition. There was no significant difference in the distribution of IgG, C3, and C1q deposition in immunofluorescence between the two groups of diseases. The distribution of IgM, C4, and Fibrogen deposition in the IgAN group was significantly lower than that in the IgAVN group [41 (17.3%) vs. 58 (30.5%), 6 (2.5%) vs. 26 (13.7%), 6 (2.5%) vs. 26 (13.7%)], and the difference was statistically significant (p = 0.003). There was no statistically significant difference in deposition type in IgA+C3, IgA+IgM/C3, IgA+IgG. However, the IgAN group was significantly higher in IgA+IgM+IgG/C3 than the IgAVN group [39 (16.5%) vs. 18(9.4 %)] (p = 0.03). The details were listed in the Table 4. 

**Table 4:** Renal pathological immunofluorescence manifestations and Oxford typing of renal pathology between two groups.

Index	IgAN (n = 237)	IgAVN (n = 190)	p
IgM	41 (17.3%)	58 (30.5%)	0.003
IgG	19 (8.0%)	10 (5.3%)	>0.05
IgA	237 (100%)	190 (100%)	/
C3	201 (84.8%)	158 (83.2%)	>0.05
Fibrogen	6 (2.5%)	26 (13.7%)	0.003
C1q	7 (3.0%)	7 (3.7%)	>0.05
Deposition type			
IgA+C3	68 (28.7%)	63 (33.2%)	>0.05
IgA+IgM/C3	125 (52.7%)	105 (55.3%)	>0.05
IgA+IgG	5 (2.1%)	4 (2.1%)	>0.05
IgA+IgM+IgG/C3	39 (16.5%)	18 (9.4%)	0.03
M (0/1)	138 (58.2%)/99 (41.8%)	114 (60.0%)/76 (40.0%)	>0.05
E (0/1)	196 (82.7%)/41 (17.3%)	124 (65.3%)/64 (34.7%)	0.003
S (0/1)	153 (64.6%)/84 (35.4%)	148 (78%)/42 (22%)	0.003
T (0/1/2)	105 (44.3%)/98 (41.4%)/34 (14.3%)	173 (91.1%)/15 (7.9%)/2 (1.0%)	0.003
C (0/1/2)	181 (76.4%)/32 (13.5%)/24 (10.1%)	119 (62.6%)/40 (21.1%)/31 (16.3%)	0.02

Oxford classification of renal pathology results showed that mesangial cells (M), endothelial hyperplasia (E), segmental glomerulosclerosis (S), renal tubular atrophy (T) and crescent formation (C) in the two groups were statistically analyzed. There was no significant difference in mesangial cells (M) between the two groups. The ratio of S1 and T1/T2 in the IgAN group was significantly higher than that in the IgAVN group [84 (35.4%) vs. 42 (22%), 98 (41.4%)/34 (14.3%) vs.15 (7.9%)/2 (1.0%)], while the ratio of crescent formation C2/C1 and E1 in the IgAN group was lower than that in the IgAVN group [32 (13.5%)/24 (20.1%) vs. 40 (21.1%)/31(16.3%), 41(17.3%) vs. 64 (34.7 %)]. The details were listed in the Table 4. 

## 4. Discussion

IgAN and IgAVN were respectively one of the common primary and secondary glomerular diseases in pediatrics [12,13]. IgAN and IgAVN were characterized by mesangial IgA deposition in renal tissue immunopathology, which cannot be distinguished by renal pathology. Therefore, some scholars have proposed that IgAN was IgAVN without skin rash [14], and both of them have common pathogenesis [15]. But are they the same disease? Some studies had explored the clinical manifestations, pathology, diagnostic and prognostic markers of IgAN and IgAVN, so as to explore the differences. But in the absence of large studies, including adults and children from different geographical part of the world, suffering from IgAN or IgAVN with or without renal impairment, it is not yet possible to conclude on their differences or similarity in terms of prognosis and sensitivity to treatment [16]. In this study, the clinical manifestations and pathology of IgAN and IgAVN were compared to find the differences and provided the basis for diagnosis and treatment. This study showed that in the demographic data, there was no significant difference in the median age, age distribution, sex and BMI of children with IgAN and IgAVN. The median course of IgAN was significantly longer than that of IgAVN. IgAN was insidious and some children had asymptomatic onset. However, about 75% of children with IgAV had kidney damage within 4 weeks of onset, and more than 95% of children with IgAV had kidney damage within 6 months [17]. With the improvement of medical conditions, patients’ awareness of follow-up visits has increased, shortening the median course of IgAVN.

In this study, the two groups of diseases had similar clinical manifestations, and the same clinical classification methods were used. The clinical types of the IgAN group were mainly hematuria proteinuria and nephrotic syndrome, and the two types accounted for more than 2/3 of the total cases. In the IgAVN group, hematuria proteinuria and nephrotic syndrome were also the most common, accounting for 55.8% of the total cases. The two types accounted for 55.8% of the total number of cases. IgAN in nephrotic syndrome type cases and its constituent ratio were significantly higher than IgAVN, which suggested that IgAN was more serious than IgAVN. The main clinical types of the two groups of diseases were quite different from domestic studies. In the two groups of diseases, chronic nephritis, isolated hematuria and nephrotic syndrome was the most common [18–20]. In this study, isolated proteinuria was found in IgAN and IgAVN clinical classification, which accounted for 3 cases (1.3%) and 20 cases (10.5%) respectively, which was similar to some domestic studies [20]. Although in this study the research objects were the first diagnosed cases in our hospital, there were also repeated visits outside the hospital, both types of cases have chronic nephritis. The main clinical types of this study were different from those of other research centers. The reasons were as follows: (1) the indications of renal puncture were different; (2) the level of diagnosis and treatment in the hospital and the compliance of patients were different; (3) there may be regional differences due to small sample size and selection bias.

For the pathological types of IgAN and IgAVN, the results of this study showed that type III and type II were more common, and the difference in pathological grading between different clinical types was statistically significant. The reasons may be as follows: (1) Clinical classification and pathological grading were not parallel; (2) In laboratory indicators, urine red blood cell count, urine protein, and blood pressure were involved in the disease and lead to different levels of renal pathological changes; the pathological changes of IgAN and IgAVN were similar. Immunofluorescence is mainly characterized by IgA deposition in the mesangial area of the glomerulus or the deposition of immune complexes dominated by IgA deposition, and it can also be manifested as mesangial hyperplasia and crescent formation. In this study, inflammatory cell infiltration was more common in the IgAN group than in the IgAVN group, and tubule atrophy and loop necrosis in the IgAN group were more obvious than those in the IgAVN group, suggesting that the degree of chronicity of IgAN renal pathology was obvious, and IgAVN was more serious in endothelial hyperplasia and crescent formation than IgAN. IgAVN showed an acute pathological process, which can be reversed by active and effective treatment. This was consistent with domestic and foreign reports [21,22].

This study also carried out renal pathological assessment on the IgAN group and IgAVN group according to Oxford classification, and concluded that M1, E1, S1, T1/2, and C1/2 were relatively common in IgAVN. Among them, endothelial cell proliferation and crescent formation are the most common, which was consistent with the results of Koji Inagaki et al. in Japan [23]. This suggested that the five classification indicators of MEST-C of Oxford classification may be applied to IgAVN. Not only can the prognosis of IgAVN be judged according to the pathological type, but also the primary and secondary glomerulonephritis dominated by IgA can adopt a unified standard. In this study, the formation of IgAVN crescent was more serious than IgAN. However, the overall kidney damage and renal function of IgAN were worse. Crescent may cause glomerular scarring, which was related to decreased renal function. However, this damage may be short-lived and can be reversal. The proportion of crescents in Oxford classification was the sum of large and small crescents, not the large or small crescents alone, which weakens its independent predictive effect, so it may not be an indicator of long-term prognosis. Yu Lei et al. [24] found that there was a strong correlation between the degree of renal tubule-interstitial lesions and prognosis. IgA has a poor prognosis and severe interstitial damage is one of the reasons. In this study, the ratio of S1 and T1/2 in the IgAN group was significantly higher than that in the IgAVN group, and the inflammatory cell infiltration was more obvious, which was consistent with the study of Yu Lei et al.

Leukocyte and hemoglobin in IgAN group were significantly lower than those in IgAVN group, and serum creatinine was significantly higher than those in IgAVN group; the clinical manifestations of children in IgAN group were more serious. The renal pathology of children in IgAN group is mainly glomerulosclerosis and renal tubular atrophy. The renal pathology of children in IgAVN group is endothelial hyperplasia and crescent formation. The renal pathological changes of children in IgAN group are also more serious than those in IgAVN group. The clinical and laboratory indexes are consistent with the renal pathological manifestations, and the two may be related. The more specific relationship between the clinical and laboratory indexes are consistent with the renal pathological manifestations needs further research.

Limitations: First, this was a retrospective study, in which some cases were eliminated due to incomplete data, which had a certain impact on the results. Second, physicians had different perception of the indications for renal puncture. Third, the two clinical entities had different classification methods: IgAVN’s International Research Classification of Kidney Diseases in Children (ISKDC) classification and IgAN’s Lee classification. The measurement standards were not unified. When comparing the pathological grading of the two groups of diseases in the same clinical type, the conclusion was different from the actual. Fourth, although current studies had found that almost all children with IgAN have more severe clinical diagnosis than IgAVN in all age groups, but the relationship between the severity of diagnosis and pathological types cannot be investigated. Fifth, lack of comparison of final outcomes, including hypertension, GFR and ESRD (end stage renal disease) or dialysis necessity. This may cause the result analysis to be not deep enough and the conclusion to be not perfect.

Conclusion: The clinical manifestations of IgAN and IgAVN were similar, but the onset of IgAN was hidden and the clinical manifestations were relatively serious. Clinical manifestations was mainly glomerulosclerosis and renal tubular atrophy. IgAVN was characterized by acute onset and good renal function. Clinical manifestations was dominated by endothelial hyperplasia and crescent formation. These differences do not support the hypothesis that the two diseases are the same. 

## Ethical considerations

This study was conducted in accordance with the declaration of Helsinki. This study was conducted with approval from the Ethics Committee of The Second Hospital of HeBei Medical University. Written informed consent was obtained from all parents/local guardians.
